# Impact of the *etr1-1* Mutation, Impairing Ethylene Sensitivity, on Hormonal Status and Growth of *Arabidopsis thaliana* Under Salinity Stress

**DOI:** 10.3390/cells14242003

**Published:** 2025-12-16

**Authors:** Anna Sevostyanova, Alla Korobova, Guzel Akhiyarova, Igor Ivanov, Guzel Kudoyarova

**Affiliations:** Laboratory of Plant Physiology, Ufa Institute of Biology, Ufa Federal Research Centre of the Russian Academy of Sciences, Pr. Oktyabrya, 69, 450054 Ufa, Russia; anka.sevostyanova@yandex.ru (A.S.); akhiyarova@rambler.ru (G.A.); i_ivanov@anrb.ru (I.I.)

**Keywords:** *Arabidopsis thaliana*, *etr1-1*, ethylene, ABA, IAA, cytokinins, salinity, growth, immunolocalisation

## Abstract

The role of ethylene in the adaptation of *Arabidopsis thaliana* to salt stress induced by 150 mM NaCl is investigated. The responses of wild-type (Columbia, WT) plants and ethylene-insensitive *etr1-1* mutants to short-term daily salt treatments were compared. Parameters analyzed included growth, water status, chlorophyll content, and hormone levels (ABA, IAA, cytokinins) using ELISA and immunohistochemistry. The results revealed that in the WT, salt stress induced hormonal redistribution: accumulation of ABA, IAA, and zeatin in shoots, accompanied by decreased ABA in the root tips and cytokinins in the whole roots. These hormonal changes were associated with stomatal closure, maintained leaf hydration, and inhibition of root growth. The inhibition of root growth may contribute to reduced uptake of toxic ions from the environment. In contrast, *etr1-1* mutants exhibited no changes in hormonal status, failed to close stomata—leading to decreased leaf water content—and showed a sharp decline in chlorophyll content accompanied by suppressed shoot growth. The conclusions emphasize that ethylene sensitivity is essential for initiating adaptive hormonal rearrangements that coordinate growth and stomatal responses to mitigate the effects of salt stress.

## 1. Introduction

Salt stress represents a major abiotic constraint significantly impairing plant growth and agricultural productivity worldwide. It disrupts cellular homeostasis by inducing ionic imbalance, osmotic stress, and secondary oxidative damage, leading to severe growth retardation [1] often governed by phytohormones.

Ethylene serves as a key regulator of plant growth, development, and stress responses. Salinity triggers a rapid increase in ethylene production [2]. However, ethylene’s role in plant adaptation to salinity appears complex. For instance, tobacco plants (*Nicotiana tabacum*) overexpressing the ethylene receptor NTHK1 exhibited salt-sensitive phenotypes, whereas application of 1-aminocyclopropane-1-carboxylic acid suppressed this salt sensitivity [3].

ETR1 represents one of five known ethylene receptors. In the absence of ethylene, ETR1 activates the protein kinase CTR1, which subsequently inhibits ethylene response. Ethylene binding to ETR1 suppresses CTR1 activity, leading to derepression of ethylene signaling [4,5]. NaCl exposure reduces expression of the ethylene receptor ETR1 in *Arabidopsis* [6].

The *etr1-1* mutants are characterized by ethylene insensitivity [7]. These mutants demonstrated enhanced sensitivity to osmotic (200 mM or higher mannitol) and salt stress (50 mM NaCl or higher) during germination and seedling development [8].

Ethylene is believed to enhance salt tolerance by activating antioxidant systems to eliminate reactive oxygen species [9] and regulating ion transport to maintain sodium and potassium homeostasis [10]. Ethylene is considered a “master regulator” of plant salt tolerance [11]. Its mechanism of action involves intricate interactions with other hormones [12], as ethylene often functions as a “conductor” of hormonal orchestration through cross-talk with other hormone signaling pathways [13]. By cooperating with other hormones, abscisic acid (ABA), auxins and cytokinins, ethylene triggers mechanisms coordinating plant growth and development in response to abiotic stresses [14]. However, the details of this interaction under salinity conditions remain understudied and they were investigated in the present work.

Our previous research demonstrated that the effect of indole-3-acetic acid (IAA) on root elongation was absent in ethylene-insensitive *Arabidopsis* mutants [15], indicating ethylene’s role in mediating auxin action in plants. However, these investigations were conducted under non-saline conditions.

Here, we performed a comparative analysis of IAA levels as well as that of ABA and cytokinins and their distribution between shoots and roots in ethylene-insensitive *etr1-1* mutants and WT Columbia plants under salinity stress. The use of the immunohistochemical method made it possible to detect ABA in the cells of root tips and leaves, as well as to identify hormonal reactions at the cellular level. We also compared growth responses to salinity between these genotypes to elucidate the potential dependence of stress adaptation on plant ethylene sensitivity. The goal of this research was to study how changes in the concentration and distribution of plant hormones between shoots and roots and their cells are related to a plant’s sensitivity to ethylene and how this interaction affects the plant’s response to salt stress.

## 2. Materials and Methods

### 2.1. Experimental Design

Experiments were performed with *Arabidopsis thaliana* ecotype Columbia as the wild-type (WT) and the ethylene-insensitive mutant *etr1-1*. Sterilized seeds were imbibed and cold-stratified for 3 days at 4 °C on moist filter paper in Petri dishes. Following stratification, seeds were sown in pots with sand substrate, pre-saturated with a ten-fold diluted Hoagland-Arnon solution (further–H-A solution) at a density of four seeds per 100 mL pot. The nutrient concentration was optimized for Arabidopsis biomass production in prior dose–response experiments. Plants were cultivated in a controlled-environment growth chamber (Sanyo, Osaka, Japan) programmed for a 16-h/8 h light/dark cycle, diurnal temperatures of 23 °C/19 °C, 80% relative humidity, and a light intensity of 120 μmol m^−2^ s^−1^ PPFD. Substrate water content was maintained at 65–70% water-holding capacity post-emergence. Commencing on the 10th day after sowing, plants were daily supplied with 2 mL of the nutrient solution. To achieve synchronous germination despite the delayed phenotype of the *etr1-1* mutant [7], the onset of its cold stratification was scheduled two days before that of the WT.

**Figure A1:**
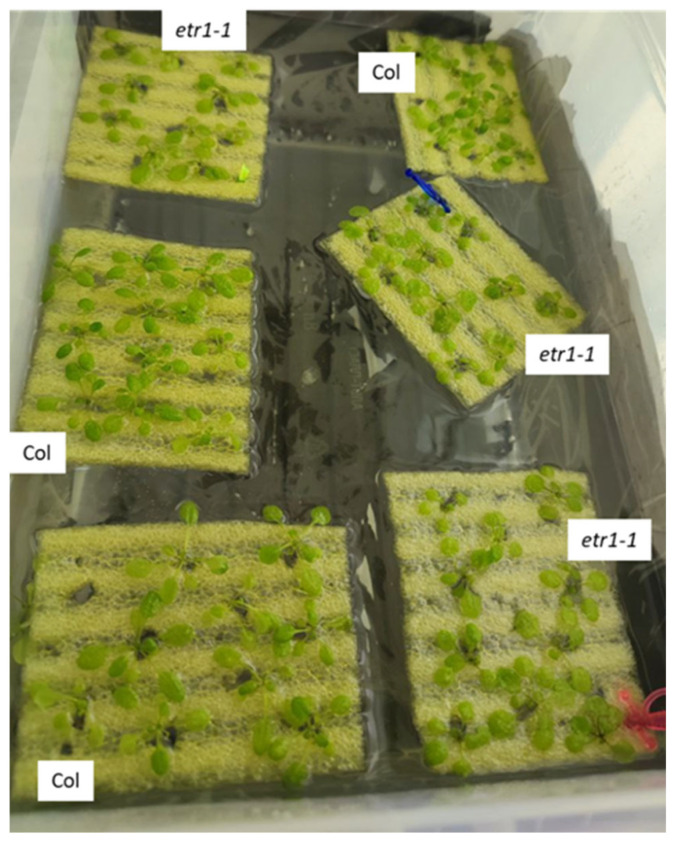
24-day-old *Arabidopsis thaliana* plant of the WT ecotype Columbia and ethylene-insensitive *etr1-1* mutants, supplied with a H-A solution supplemented with NaCl. For three days, plants were exposed to a nutrient solution containing sodium chloride (final concentration 150 mM) for three hours.

Three-week-old *Arabidopsis* plants were transferred to a hydroponic system, whereby they were secured on floating rafts (10 plants per a raft) over an aerated H-A solution. A salt stress treatment was initiated by supplementing the H-A solution with sodium chloride to a final concentration of 150 mM in half of the containers (Figure A1). This concentration was determined to be effective in preliminary dose–response assays. *Arabidopsis thaliana*, a glycophyte, is notably sensitive to saline conditions [16]. To effectively capture the cumulative physiological effects of salinity, which develop over several days, plants were exposed to salt treatment for 3 h daily. This experimental design also served to mimic natural soil conditions where salts are distributed heterogeneously, and root systems can transiently encounter localized saline patches [17].

Plant tissues were harvested and fixed for subsequent phytohormonal analysis immediately following the final NaCl treatment on the third experimental day. Phytohormone profiling included quantitative analysis of abscisic acid (ABA), indole-3-acetic acid (IAA), and cytokinins using enzyme-linked immunosorbent assay (ELISA). In addition, immunohistochemical localization of ABA was performed in root tips. Concurrent physiological assessments included stomatal conductance and chlorophyll content. Growth characteristics were assessed the day after the samples were taken for hormonal analysis

For each plant we measured root length, lateral root number and length, shoot and root biomass and their ratio. Also, leaf tissue water content was calculated from gravimetric measurements of shoot fresh and dry mass.

### 2.2. Measurement of Stomatal Conductance and Chlorophyll Content

Stomatal conductance measurements were performed with an LI-600 porometer/fluorometer (LI-COR Biosciences, Lincoln, NE, USA). Concurrently, leaf chlorophyll content was assessed non-destructively using a DUALEX SCIENTIFIC+ meter (FORCE-A, Paris, France).

### 2.3. Purification, Concentration, and Quantification of Hormones

For hormone extraction, shoots and roots of *Arabidopsis* plants were homogenized in 80% ethanol and incubated overnight at 4 °C. The aqueous supernatant, obtained after centrifugation, filtration and ethanol evaporation, was divided into two equal aliquots. One aliquot was used for purification and concentration of abscisic acid (ABA) and indole-3-acetic acid (IAA) according to established protocols [18,19]. After adjusting the pH to 2.5 with HCl the extract was partitioned with diethyl ether. Subsequently, free IAA and ABA were transferred from the organic phase into an equal amount of 1% sodium hydrocarbonate (pH 7–8), while methylated hormones were retained in the organic phase. Readjusting the pH of the aqueous phase to 2.5 and re-extraction with ether, gave the secondary ether extract, which was methylated and solvent was evaporated. The procedure released the samples containing free hormones from their methylated forms and therefore concentration of free hormones was immunoassayed. The final residue was dissolved in 100 µL of 80% ethanol, and a portion of this solution was used for ELISA with specific anti-ABA or anti-IAA antibodies. Conjugation of IAA and ABA to proteins using carbodiimide and immunization schemes were described by Veselov et al. [19]. For the enzyme immunoassay of auxin concentration, we used a protein conjugate with IAA, and for measuring the ABA concentration, a protein conjugate with ABA was used. There was no cross-reaction between IAA antibodies and ABA, nor between ABA antibodies and IAA. The specificity of the enzyme immunoassay was confirmed by low cross-reactivity of antibodies with hormone precursors and derivatives as well as by the coincidence of chromatographic distribution of immunoreactivity with the position of hormonal standards detected in the purified hormonal samples [19]. No immunoreactivity was detected in the eluate with a Rf corresponding to the position of the methylated standard. Recovery of IAA and ABA was calculated using the partition constants of hormones between aqueous and organic phases and was found to be about 90%. Reliability of immunoassay of ABA was also confirmed by comparison of the results of the method with those of the physicochemical assay (HPLC-MS) [18].

The second aliquot of the aqueous supernatant was subjected to solid-phase extraction using a C18 sorbent cartridge (900 mg Sorbent per Cartridge, Waters, Milford, MA, USA) [20]. Following solvent evaporation, the residue was dissolved in 20 µL of 80% ethanol and subjected to thin-layer chromatography (TLC) on silica gel 60 F-254 plates (Merck; 50 × 200 × 0.25 mm) using a 2-butanol:14M NH_4_OH:H_2_O (6:1:2 *v*/*v*, upper phase) solvent system. This protocol effectively separated zeatin nucleotide (Rf 0–0.1), zeatin glucosides (Rf 0.1–0.2), zeatin riboside (Rf 0.4–0.5), and zeatin (Rf 0.6–0.7) [21].

Cytokinin-containing zones, identified by co-migration with authentic standards, were eluted with 0.1 M phosphate buffer (pH 7.4) for 12 h. The eluates were serially diluted and directly assayed via enzyme-linked immunosorbent assay (ELISA) using antibodies against trans-zeatin riboside. Conjugation of zeatin riboside to protein was performed using periodate reaction. Preparation of conjugate and immunization schemes were as per Veselov et al. 1998 [22]. These antibodies exhibit high specificity for trans-zeatin, its riboside, nucleotide, and 9-N-glucoside, with minimal cross-reactivity toward dihydrozeatin and isopentenyladenine (iP) derivatives [20]. Validation of the method demonstrated >90% recovery for standards (no more than 5% loss of cytokinins immunoreactivity both at the stage of solid-phase extraction using a cartridge with a C18 sorbent and elution from silica gel 60 F-254 plates), and immunoassay reliability was confirmed by dilution tests, chromatographic immunoreactivity profiling, and correlation with LC-MS data [20].

Phytohormones were quantified using a competitive enzyme-linked immunosorbent assay (ELISA) with specific antibodies, as previously described [18]. Briefly, microplate wells were coated with a hormone-protein conjugate in phosphate-buffered saline (PBS, pH 7.4). After incubating and washing, wells were incubated with a mixture of hormone standards or plant extracts and specific anti-hormone antiserum. In this competitive format, endogenous hormones and the immobilized conjugate compete for binding sites on the primary antibodies. Following another wash step to remove unbound serum, wells were incubated with peroxidase-conjugated secondary antibodies against rabbit immunoglobulins. The enzyme reaction was initiated by adding o-phenylenediamine substrate in phosphate-citrate buffer (pH 5.5) containing 0.03% H_2_O_2_. Optical density was determined at 492 nm using a UNIPLAN AIFR-01 microplate spectrophotometer (PIKON, Moscow, Russia).

### 2.4. Immunohistochemical Localization of Abscisic Acid

Root tips of the WT and mutant plants were cut to a length of 5 mm with a sharp razor blade in a minimum volume of water. Root and leaf tissues were fixed in freshly prepared fixative solution (2% 1-ethyl-3-(3-dimethylaminopropyl) carbodiimide, 0.5% Triton X-100, and 0.5% Tween-20) on 1/10 MTS buffer (50 mM PIPES, 5 mM MgSO_4_·7H_2_O, 5 mM EGTA, pH 7.2) [23]. Treatment of root tip sections with carbodiimide promoted conjugation of both IAA and ABA with plant tissue proteins/This prevented their washout during ethanol dehydration (see below) and enabled recognition by antibodies obtained by immunizing rabbits with a carbodiimide-prepared hormone–protein conjugate. To optimize fixative penetration, root tissues were vacuum treated during the first 60 min of fixation. Postfixation was then performed in 3% paraformaldehyde solution (Riedel de Haen, Seelze, Germany) on 1/10 MTS buffer for 12 h at 4 °C.

To obtain root tissues, sections of the root samples were washed in 1/10 MTS buffer, dehydrated in increasing ethanol concentrations and embedded in JB-4 resin (Sigma, St. Louis, MO, USA) according to the manufacturer’s instructions using special molds. 1.5-µm-thick sections were obtained on an HM 325 rotary microtome (MICROM Laborgeräte, Walldorf, Germany) [24].

Before applying primary antibodies, 50 µL of blocker solution (0.2% gelatin and 5% BSA) on 1/10 MTS buffer were applied to each section (for 1 h at 28 °C in a humidified chamber). 20 μL of rabbit polyclonal antibodies to abscisic acid (diluted 1:100 in 1/10 MTS buffer) were applied to each section and incubated overnight at 4 °C overnight. Then the slides were washed in a solution (0.5% blocker in 1/10 MTS buffer) three times for 10 min. After blocking, sections were coated with the Alexa488-labeled goat anti-rabbit secondary antibodies (Invitrogen, Rockford, IL, USA). The secondary antibodies were diluted (1:1000, according to the instructions) in a 1/10 MTS buffer and applied at a volume of 20 μL to each section. For incubation of sections with secondary antibodies, slides were placed in a humid chamber and maintained at 28 °C for 2 h. A final wash in the solution was performed 10 times for 3 min, followed by rinsing with water. To stain cell walls in root tissue sections, SCRI Renaissance (SR2200) dye at a dilution of 1:2000 was applied and maintained for 1 h at room temperature. At the final stage, sections were mounted in ProLong Glass antifade (Invitrogen, Waltham, MA, USA) and left overnight at room temperature.

Fluorescence images were acquired using an Olympus FV3000 Fluoview (FV31-HSD) confocal laser scanning microscope (Olympus, Tokyo, Japan) with excitation laser wavelengths of 488 nm (for Alexa488) and 405 nm (for SR2200). Fluorescence emission was recorded in the following ranges: 488–588 nm (for the 488 laser) and 407–459 nm (for the 405 laser). The specificity of staining of the hormones studied was confirmed by the absence of fluorescence in samples treated with serum containing no antibodies to ABA. Averaged fluorescence per one pixel registered with the confocal microscope was estimated with ImageJ version 1.53 software (National Institutes of Health, Bethesda, MD, USA, https://imagej.net/ij/download.html, accessed on 27 May 2020).

### 2.5. Statistical Analysis of Data

A group of 20 plants served as a biological replicate for determining hormone levels using ELISA. Three independent experiments were conducted, with three biological replicates in each variant (*n* = 9). For immunolocalization, one section from one plant served as the biological replicate.

The data were statistically processed using Statistica version 10 software (Statsoft, Moscow, Russia). In figures and tables, data are presented as mean ± SE. The significantly different means were revealed by *t*-test or ANOVA followed by Duncan’s test (*p* ≤ 0.05).

## 3. Results

**Figure 1:**
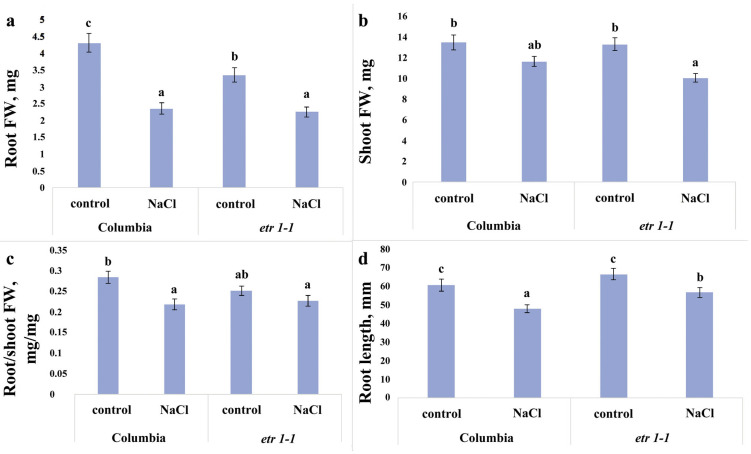
Root (**a**) and shoot (**b**) biomass, root-to-shoot ratio (**c**), and average primary root length (**d**) of 25-day-old *Arabidopsis thaliana* plants of the WT ecotype Columbia and ethylene-insensitive *etr1-1* mutants, grown on H-A solution without salt (control) or supplemented with NaCl. For four days, plants were exposed to a nutrient solution containing sodium chloride (final concentration 150 mM) for three hours. Statistically different means (*n* = 40) are indicated by different letters (*p* < 0.05, ANOVA + Duncan’s test).

A four-day exposure of the plants from the WT Columbia line and the ethylene-insensitive *etr1-1* mutants to the sodium chloride-containing medium resulted in a reduction in root biomass and length in both genotypes (Figure 1a,d). This effect was more pronounced in Columbia plants compared to the mutants. Specifically, root biomass under salinity stress decreased by nearly half in the WT line, whereas the mutant plants exhibited only a 30% reduction. The root length of sodium chloride-treated plants was 20% and 15% lower than that of the control groups for Columbia and *etr1-1*, respectively. Comparison of root biomass in control plants not subjected to salinity revealed that the mutant plants were inferior to Columbia plants in this parameter (Figure 1a).

Salinity did not cause a substantial decrease in shoot biomass accumulation in Columbia plants, whereas it inhibited this process in mutant plants (Figure 1b). Consequently, unlike Columbia, the mutant plants did not exhibit a decrease in the root-to-shoot ratio (Figure 1c), indicating no relative inhibition of root growth.

The number of lateral roots per plant was not affected by salinity in either the WT line or the ethylene-insensitive mutants (Table 1). However, a reduction in both the average length of a single lateral root and the total lateral root length per plant was recorded in Columbia plants (Table 1).

**Figure 2:**
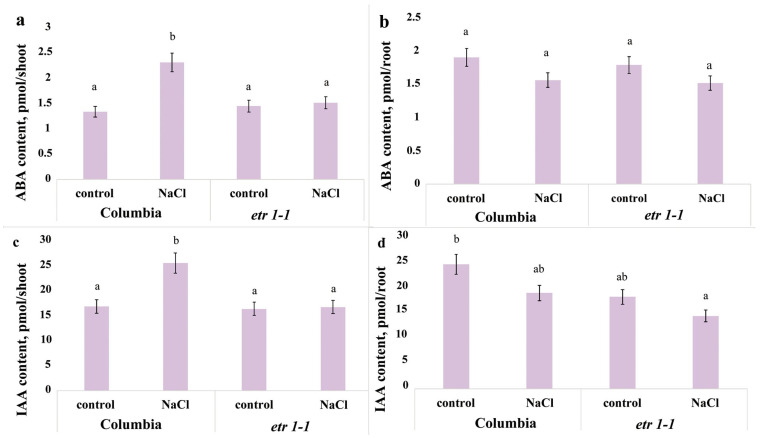
Content of ABA (**a**,**b**) and IAA (**c**,**d**) in shoots (**a**,**c**) and roots (**b**,**d**) of single 24-day-old *Arabidopsis thaliana* plant of the WT ecotype Columbia and ethylene-insensitive *etr1-1* mutants, supplied with a H-A solution without salt or supplemented with NaCl. For three days, plants were exposed to a nutrient solution containing sodium chloride (final concentration 150 mM) for three hours. Statistically different means (*n* = 9) are indicated by different letters (*p* < 0.05, ANOVA + Duncan’s test).

Following three exposures to the H-A solution supplemented with sodium chloride quantitative immunoassay showed an approximately two-fold accumulation of ABA and IAA in the shoots of Columbia plants, whereas no significant changes in the content of these hormones occurred in the shoots of mutant plants, (Figure 2a,c). In the roots of both lines, no statistically significant changes in hormone content were detected under salt stress (Figure 2b,d).

**Figure 3:**
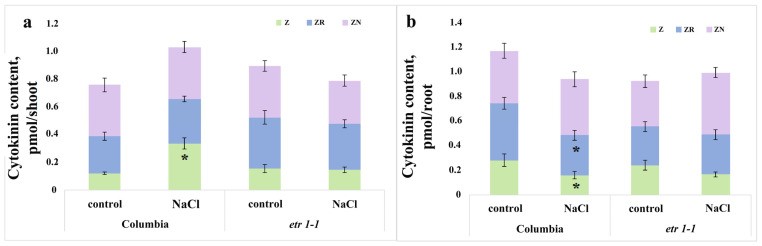
Content of zeatin (Z), its riboside (ZR), and nucleotide (ZN) in shoots (**a**) and roots (**b**) of single 24-day-old *Arabidopsis thaliana* plant of the WT ecotype Columbia and ethylene-insensitive *etr1-1* mutants, supplied with a H-A solution without salt or supplemented with NaCl. For three days, plants were exposed to a nutrient solution containing sodium chloride (final concentration 150 mM) for three hours. An asterisk indicates mean hormone content values under salinity that are statistically different from the control plant values (*n* = 9; *p* < 0.05, *t*-test).

Measurement of cytokinin content revealed a significant difference only in zeatin levels in salt-treated Columbia plants compared to controls. Specifically, we recorded an increase in zeatin and its riboside in leaves, and conversely, a decrease in their content in roots (Figure 3).

**Figure 4:**
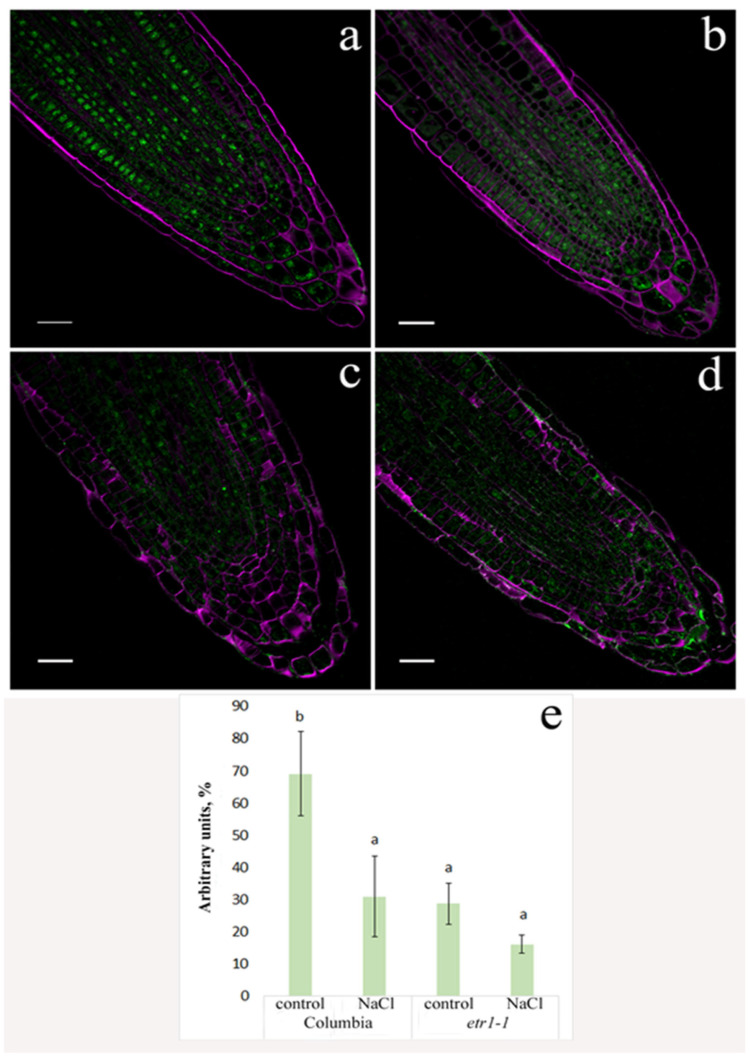
Immunohistochemical staining of ABA (green color) in root tips of 24-day-old *Arabidopsis thaliana* plants of the WT ecotype Columbia (**a**,**c**) and ethylene-insensitive *etr1-1* mutants (**b**,**d**), supplied with a H-A solution without salt (**a**,**b**) or supplemented with NaCl (**c**,**d**). Intensity of fluorescence for ABA (**e**); means ± s.e., arbitrary units, maximal fluorescence taken as 100%, minimal-as 0%. For three days, plants were exposed to a nutrient solution containing sodium chloride (final concentration 150 mM) for three hours. Purple staining—SR2200 Renaissance; green—Alexa488. Scale bar—20 µm. Statistically different means (*n* = 9) are indicated by different letters (*p* < 0.05, ANOVA + Duncan’s test).

Using an immunohistochemical method, we detected a decrease in ABA levels in the root tip cells of Columbia plants (Figure 4a,c,e). In contrast, no changes in hormone-specific fluorescence were observed in mutant plants (Figure 4b,d,e).

**Figure 5:**
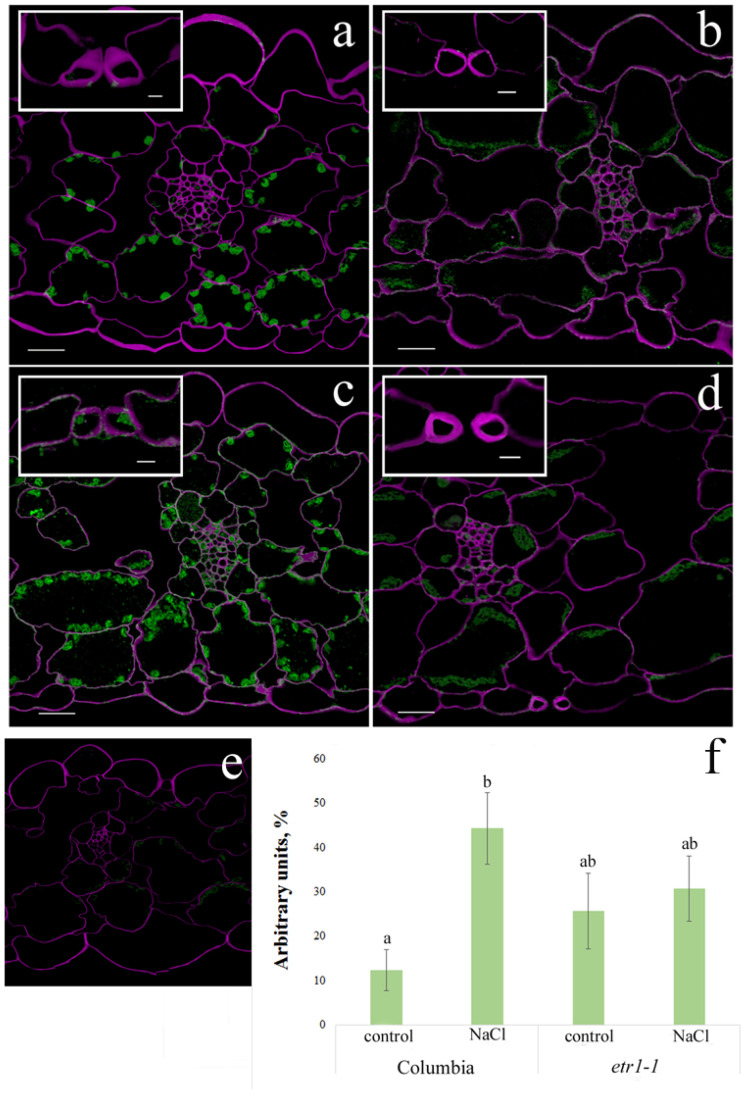
Immunohistochemical staining of ABA (green color) in leaves of 24-day-old *Arabidopsis thaliana plants* of the WT ecotype Columbia (**a**,**c**) and ethylene-insensitive *etr1-1* mutants (**b**,**d**), supplied with a H-A solution without salt (**a**,**b**) or supplemented with NaCl (**c**,**d**); treatment with non-immune serum (**e**). Intensity of fluorescence for ABA (**f**); means ± s.e., arbitrary units, maximal fluorescence taken as 100%, minimal as 0%. For three days, plants were exposed to a nutrient solution containing sodium chloride (final concentration 150 mM) for three hours. Purple staining—SR2200 Renaissance; green—Alexa488. Scale bar—20 µm. Higher magnification images of stomatal guard cells are inserted in each figure. Statistically different means (*n* = 9) are indicated by different letters (*p* < 0.05, ANOVA + Duncan’s test).

Immunohistochemical staining for ABA in leaf cells of the WT line and ethylene-insensitive mutants confirmed the results obtained by enzyme-linked immunosorbent assay. In Columbia leaves, exposure to sodium chloride induced an accumulation of this hormone, whereas in *etr1-1* leaves, salinity did not alter ABA levels (Figure 5). Green coloring corresponding to ABA presence was most pronounced in leaf sections of the salt-stressed Columbia plants. Fluorescence of chloroplasts noticeable on the leaf sections was absent, when non-immune serum was used instead of antiserum against ABA, confirming specificity of ABA immunolocalization. The results are consistent with those of McAdam and Brodribb, who reported on the presence of ABA in chloroplasts [25].

**Figure 6:**
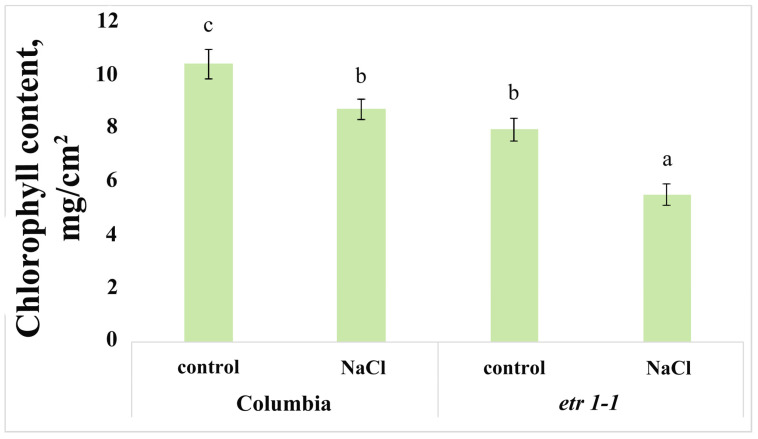
Chlorophyll content in 24-day-old *Arabidopsis thaliana* plants of the WT ecotype Columbia and ethylene-insensitive *etr1-1* mutants, supplied with a H-A solution without salt or supplemented with NaCl. For three days, plants were exposed to a nutrient solution containing sodium chloride (final concentration 150 mM) for three hours. Statistically different means (*n* = 40) are indicated by different letters (*p* < 0.05, ANOVA + Duncan’s test).

Chlorophyll content decreased under salinity stress in both genotypes. However, the reduction was more pronounced in mutant plants compared to Columbia (17% and 30% decrease relative to their respective controls for Columbia and *etr1-1*, respectively). Mutant plants not exposed to sodium chloride exhibited significantly lower chlorophyll levels than control Columbia plants (Figure 6). Consequently, under saline conditions, the chlorophyll content in mutants was only 50% of that measured in control Columbia plants.

Under the influence of sodium chloride, WT Columbia plants exhibited a reduction in stomatal conductance, while shoot water content remained at the level of control plants. In contrast, ethylene-insensitive *etr1-1* plants displayed the opposite response: no changes in stomatal conductance were observed, but a decrease in shoot tissue hydration occurred (Table 2).

## 4. Discussion

### 4.1. Alterations in Growth and Hormonal Status of the Wild-Type Arabidopsis thaliana Plants Under Sodium Chloride Salinity

Wild-type Columbia plants exhibited an adaptive response to the introduction of sodium chloride into the nutrient medium, manifested by an inhibition of root growth relative to the shoot. These plants showed a significant reduction in root biomass accumulation, while shoot growth was not inhibited during the experiment (Figure 1). The inhibition of root growth may play an important role in reducing uptake of toxic ions into the plant. This conclusion was drawn by Fedoreeva et al. [26] based on their study of the effects of sodium chloride salinity on wheat plants. Their data indicated that salinity in durum wheat led to the inhibition of root growth (both biomass and length), which was associated with reduced uptake of toxic sodium ions.

The shoot biomass of plants treated with sodium chloride did not lag behind that of control plants during the experiment (Figure 1), despite the elevated ABA level (Figure 2 and Figure 5). Given that cytokinins promote shoot growth [27], the maintenance of shoot biomass accumulation observed in our study could be associated with the increased content of the cytokinin zeatin in the shoots (Figure 3). In the roots, we recorded a decrease not only in zeatin but also in its riboside. It can be hypothesized that salinity induced a redistribution of these hormones in favor of the shoots. The accumulation of ABA, IAA, and zeatin in the shoot (Figure 2, Figure 3 and Figure 5) may have been significant for maintaining the functional activity of the photosynthetic apparatus [28,29,30].

The reduction in root elongation observed in plants treated with sodium chloride (Figure 1) could be a consequence of the decreased ABA content in the root tips (Figure 4). Exogenous abscisic acid (ABA) is known to either stimulate or inhibit root growth, depending on its concentration and interaction with ethylene [31]. It was shown that ABA and its interaction with ethylene play an essential role in salt-modulated root growth [32]. Under water deficit conditions, increased ethylene production necessitates elevated levels of ABA to prevent ethylene-induced growth inhibition [33]. Given that ethylene production often increases in plants under salinity stress [9], the reduced ABA content in the roots of salt-stressed plants may have potentiated the inhibitory effect of ethylene on root elongation under conditions of the present experiments.

Root biomass is determined not only by the mass of the primary root but also by root branching. The reduction in fresh root mass under sodium chloride influence could be a consequence of inhibited lateral root elongation (Table 1). According to the literature, sodium chloride at concentrations of 75–150 mM sequentially inhibited primary root length, lateral root length, and the lateral root number in *Arabidopsis* plants [34]. In our experiments, we recorded inhibition of primary and lateral root elongation, whereas no change in the lateral root number occurred. Since we worked with mature plants and measured growth parameters only three days after the onset of salt exposure, which was applied for just 3 h daily, changes in the lateral root number might not have been manifested at this early stage. Furthermore, we did not detect a decrease in root IAA levels, which regulates lateral root initiation.

### 4.2. Alterations in Growth and Hormonal Status of Ethylene-Insensitive Arabidopsis thaliana etr1-1 Mutant Plants Under Sodium Chloride Salinity

In contrast to the parental Columbia line, exposure to sodium chloride inhibited shoot biomass accumulation in ethylene-insensitive Arabidopsis thaliana *etr1-1* mutants (Figure 1b). Root length and biomass also decreased, but to a lesser extent than in Columbia (Figure 1a,d). The root-to-shoot fresh weight ratio in mutants remained unchanged under salt stress (Figure 1c). This suggests that the loss of ethylene sensitivity impaired the mutant plants’ ability to fully adapt to the presence of sodium chloride in the medium. Judging by the reduction in shoot biomass (Figure 1b), *etr1-1* plants suffered more from salinity stress than Columbia plants.

No changes in ABA levels were detected in the shoots, entire roots, or root tips of the mutants (Figure 2 and Figure 4). Mutations in genes encoding ethylene receptors are dominant. Inactivation of even one of the five genes (*ETR1*, *ETR2*, *ERS1*, *ERS2*, *EIN4*) can lead to loss of ethylene sensitivity at the whole-plant level [35]. Even if ethylene production increased in mutant plants in response to sodium chloride, it could not influence growth and physiological processes. Consequently, changes in ABA levels to counteract (or not counteract, in the case of reduced ABA) ethylene action were not required.

If ABA did not accumulate in the shoots of mutant plants, what then caused the inhibition of their biomass accumulation under salinity? The sharp decline in chlorophyll content in *etr1-1* under salinity (Figure 6) likely indicates inhibition of photosynthesis, which most probably led to the reduction in shoot biomass. Experiments with ethephon, an ethylene-releasing compound, have demonstrated increased chlorophyll content [36,37]. Since ethylene can help maintain chlorophyll levels in plants, it is plausible that the decline in pigment in plants with impaired ethylene reception was directly linked to the absence of ethylene signaling. This is further supported by the observation that mutant plants not subjected to salinity contained 24% less chlorophyll than control Columbia plants (Figure 6).

In the absence of salinity, the roots of mutant plants exhibited lower biomass compared to Columbia plants (Figure 1a). We cannot explain these results based on the content of the hormones studied, as their levels in the control *etr1-1* plants did not differ from Columbia in either shoots or roots (Figure 2 and Figure 3). Root growth undoubtedly depends on the availability of photoassimilates supplied from the shoot. It has been shown that blocking photosynthesis suppresses root growth, while adding sucrose to the growth medium can rescue it [38]. In our work, we observed reduced chlorophyll content in the leaves of ethylene-insensitive mutants compared to control Columbia plants (Figure 6). It can be hypothesized that the photosynthetic rate was lower in mutant plants than in Columbia, potentially leading to their lower biomass accumulation.

The reduced root biomass accumulation in mutant plants under salinity could also be a consequence of decreased photosynthetic rates, given the sharp decline in leaf chlorophyll content we recorded. However, the inhibition of root biomass accumulation in *etr1-1* was less pronounced than in Columbia. This is not surprising, as we detected no changes in root hormone levels or lateral root length in the mutant plants. In *etr1-1* plants, sodium chloride only inhibited primary root elongation, which could be a consequence of reduced chlorophyll content.

### 4.3. Effects of Sodium Chloride Salinity on Water Parameters in COLUMBIA and Ethylene-Insensitive etr1-1 Mutant Plants

The 150 mM sodium chloride solution exerts both toxic and osmotic effects on plants, making it essential to evaluate alterations in their water parameters under these conditions.

We observed a reduction in stomatal conductance in Columbia plants subjected to salinity compared to control plants (Table 2). This response was likely a consequence of ABA accumulation in the leaves, as stomatal closure mediated by elevated ABA levels is a well-established phenomenon [39]. The increased ABA level was detected not only in whole shoots via immunoassay but also through immunolocalization in leaf cells, particularly evident in the guard cells (Figure 2 and Figure 5). Our data align with a study on barley, where salinity-induced reduction in transpiration rate was accompanied by ABA accumulation predominantly in leaf mesophyll cells [40].

The stomatal closure recorded in Columbia plants in our experiments was crucial for maintaining plant water status, as no decrease in shoot water content was detected after three salt exposure sessions (Table 2).

In contrast, exposure of mutant plants to the sodium chloride solution produced the opposite effect compared to Columbia. The *etr1-1* plants exhibited decreased shoot tissue hydration (Table 2), which is unsurprising given the absence of reduced stomatal conductance (Table 2) and the diminished water potential of the saline solution limiting water inflow from the roots. Stomata likely remained open, possibly due to the lack of an ABA signal, since no increase in its level in the leaves occurred. The absence of ABA accumulation under salinity in the ethylene-insensitive mutant indicates a role for ethylene in regulating ABA levels. Reports on ethylene’s influence on ABA synthesis are conflicting, with evidence supporting both stimulation [41] and inhibition [42] of ABA biosynthesis by ethylene.

The accumulation of ABA in Columbia leaves occurred concurrently with a decrease in this hormone’s level in root tips (Figure 2, Figure 4 and Figure 5), suggesting a potential role for ABA transport between shoot and root in regulating its concentration. Existing evidence of ethylene’s influence on auxin transport [43], combined with our results, highlights the need to investigate ethylene’s effect on the recently identified ABA transporters [44]. In *Arabidopsis*, several such transporters have been characterized to varying degrees (see reviews by [44]), among which the genes encoding ABCG25 and ABCG40 have been studied in most detail. The experiments described in this paper implicate the AtABCG25 transporter in the translocation of abscisic acid from roots to shoots, which mediates stomatal movements in *Arabidopsis*. It has been shown that this transporter is capable of carrying not only free ABA but also ABA-glucosyl ester, which can serve as a long-distance signal alongside free ABA [45]. We have not paid due attention to ABA-glucosyl ester, which should be corrected in the future.

Ethylene can induce stomatal closure [46]; however, elevated ethylene concentrations may inhibit stomatal closure triggered by abscisic acid and jasmonic acid [47,48]. Among the five ethylene receptors, only ETR1, EIN4, and ERS1 are involved in ethylene-mediated stomatal closure, as demonstrated by the impaired response to exogenous ethylene in mutants with defective ETR1, EIN4, and ERS1 function [48]. In our study, *etr1-1* mutants showed no ABA accumulation in shoots, and ethylene was likewise unable to induce stomatal closure due to the impaired function of the ETR1 ethylene receptor.

## 5. Conclusions

The four-day regimen of daily three-hour exposure to a nutrient solution containing sodium chloride adversely affected root growth in both the wild type and mutant genotypes. Root growth inhibition under salinity represents a crucial plant adaptation mechanism, as it reduces the surface area for uptake of toxic ions. However, the extent of root elongation and biomass inhibition was less pronounced in ethylene-insensitive plants compared to Columbia. Furthermore, unlike the WT, *etr1-1* mutants exhibited reduced shoot biomass accumulation. This suggests that the loss of ethylene sensitivity compromises the plants’ ability to adequately adapt to saline conditions.

The hormonal status of Columbia and *etr1-1* plants diverged significantly under salinity. WT plants accumulated ABA, IAA, and cytokinin zeatin in their shoots, while simultaneously exhibiting reduced cytokinin levels in roots and decreased ABA content in root tips. In contrast, mutant plants showed no significant changes in hormone content under our experimental conditions. Consequently, *etr1-1* plants failed to close their stomata under salinity, resulting in decreased leaf hydration. In Columbia plants, sodium chloride-induced ABA accumulation in shoots presumably led to reduced stomatal conductance, thereby maintaining leaf water content at control levels. The decreased ABA in Columbia root tips might have facilitated ethylene-mediated inhibition of root elongation. Redistribution of cytokinins toward the shoots prevented the sharp decline in chlorophyll content observed in mutants, potentially supporting shoot growth under saline stress.

The accumulation of ABA in WT shoots may have resulted from salt-induced ethylene production. This is supported by literature demonstrating that ethephon treatment, which releases ethylene, enhances ABA biosynthesis [49]. The absence of ABA accumulation in leaves of ethylene-insensitive mutants further corroborates this conclusion.

Thus, accumulation of ABA in shoots of salt-stressed Columbia plants was likely due to increased synthesis of this hormone, while changes in ABA distribution between shoots and roots suggest involvement of hormone transport. Both effects were shown to depend on sensitivity to ethylene, since they were absent in ethylene insensitive *etr1-1* mutants. Further experiments are needed to confirm the importance of ABA synthesis, transport and possibly degradation rate in the control of the level of this hormone.

**Figure 7:**
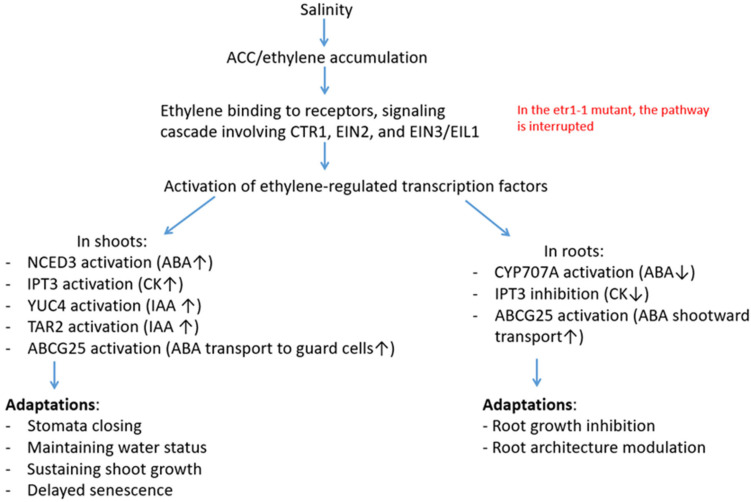
A mechanistic model summarizing proposed interactions among ethylene, ABA, auxin, and cytokinin under salinity. The arrows indicate an increase (↑) or decrease (↓) in hormone levels. *Primary signal and perception*. Salinity induces osmotic stress and ion toxicity, which serves as the primary signal for activating stress responses. In wild-type plants (Columbia), stress leads to increased production of 1-aminocyclopropane-1-carboxylic acid (ACC), an ethylene precursor, and subsequent ethylene accumulation [2]. Ethylene binds to receptors (e.g., ETR1, ETR2), which, through a signaling cascade involving CTR1, EIN2, and EIN3/EIL1, leads to the activation of ethylene-regulated transcription factors [5]. *Ethylene–ABA interaction*. Ethylene can increase the expression of key genes involved in ABA biosynthesis, such as NCED3 (9-cis-epoxycarotenoid dioxygenase 3) [49]. This explains the observed ABA accumulation in Columbia plant shoots under saline conditions. Accumulation of ABA in shoots also can occur through its active export into the vascular system via the ABCG25 transporter, which is expressed in parenchyma cells [50]. Ethylene can activate ABA catabolism in root tips through the regulation of CYP707A (ABA-8′-hydroxylase) family genes. This is evidenced by the detected expression of CYP707A (ABA-8′-hydroxylase) family genes in roots [51] and the ability of ethylene to activate the expression of these genes [52]. ABA degradation could be the cause of the observed decrease in ABA levels in Columbia root tips. ABA accumulation in leaves induces stomatal closure (via ion channel activation), which reduces water loss and maintains water status. A decrease in ABA levels in the root meristem removes its inhibitory effect on cell division, which, however, makes root growth more sensitive to the direct inhibitory effect of ethylene (leading to adaptive inhibition of elongation). *Ethylene–CK interaction*. Under saline conditions, a gradual decrease in IPT3 gene expression was found in tomato plants in the roots and an increase in it in the leaves [53]. Since ethylene production generally increases under saline conditions, it can be assumed that the increase in cytokinin content in shoots and the decrease in roots in our experiments could be due to changes in the expression of the corresponding genes. Maintaining CK levels in shoots delays leaf senescence and preserves chlorophyll content [54]. *Ethylene–Auxin interaction*. Under saline conditions, plants can accumulate IAA in shoot tissues due to the activation of biosynthesis [55]. In our experiments, ethylene-insensitive mutants did not accumulate IAA in shoots exposed to salt. This suggests the possible involvement of ethylene in modulating auxin levels in shoots. Ethylene modulates auxin transport by regulating the expression and localization of PIN proteins [56]. Under stress, this can lead to IAA redistribution, specifically to a decrease in auxin efflux from shoots to roots. High IAA levels in leaves may contribute to the maintenance of shoot growth [29] under saline conditions. *In mutant plants* (red), ethylene does not bind to the ETR1 receptor, and the signal transduction cascade is not initiated. Furthermore, no changes in hormonal balance are observed (increased levels of ABA, IAA, and zeatin in shoots, decreased levels of cytokinins in roots, and decreased ABA levels in root tips). Mutant plants with impaired ethylene reception were more susceptible to salinity than plants of the original Columbia line.

In the diagram, we presented a possible chain of events occurring in wild-type plants, from the onset of salinity to the manifestation of an adaptive response (Figure 7).

Our findings underscore ethylene’s crucial role in enhancing plant salt tolerance. They also indicate that ethylene contributes to plant adaptation to salinity both through direct effects on growth processes and by modulating the concentrations of other hormones (ABA, IAA, cytokinins).

According to literature, *Arabidopsis* mutants overproducing ethylene (due to mutation in the ETO1 gene) exhibit enhanced salt tolerance associated with increased reactive oxygen species levels and improved Na^+^/K^+^ homeostasis [10]. These findings reinforce the importance of ethylene in plant adaptation to salinity and define promising directions for our future research.

## Figures and Tables

**Table 1:** Number and total length of lateral roots per plant, and average length of a single lateral root in 25-day-old *Arabidopsis thaliana* plants of the WT ecotype Columbia and ethylene-insensitive *etr1-1* mutants, supplied with a H-A solution without salt (control) or supplemented with NaCl. For three days, plants were exposed to a nutrient solution containing sodium chloride (final concentration 150 mM) for three hours. An asterisk indicates mean growth parameter values under salinity that are statistically different from the control plant values (*n* = 40; *p* < 0.05, *t*-test).

Genotype	Treatment	Number of Lateral Roots	Total Lateral Root Length, mm	Average Length of a Single Lateral Root, mm
Columbia	Control	19 ± 3	118 ± 8	6.9 ± 0.6
	+NaCl	17 ± 2	95 ± 8 *	5.4 ± 0.4 *
*etr1-1*	Control	16 ± 3	115 ± 11	6.8 ± 0.5
	+NaCl	17 ± 4	117 ± 9	6.2 ± 0.6

**Table 2:** Shoot water content and stomatal conductance in 24–25-day-old *Arabidopsis thaliana* plants of the WT ecotype Columbia and ethylene-insensitive *etr1-1* mutants, supplied with a H-A solution without salt or supplemented with NaCl. For three-four days, plants were exposed to a nutrient solution containing sodium chloride (final concentration 150 mM) for three hours. Statistically different means (*n* = 40) for each parameter are indicated by different letters (*p* < 0.05, ANOVA+ Duncan’s test).

Genotype	Treatment	Stomatal Conductance, m^−2^ s^−1^	Shoot Water Content, %
Columbia	Control	0.45 ± 0.06 ^b^	95.7 ± 0.1 ^a^
	NaCl	0.31 ± 0.03 ^a^	95.9 ± 0.1 ^a^
*etr1-1*	Control	0.38 ± 0.05 ^ab^	96.0 ± 0.1 ^a^
	NaCl	0.44 ± 0.04 ^b^	94.1 ± 0.1 ^b^

## Data Availability

All the data are presented in this manuscript.
